# Dimethyloxalylglycine Suppresses SREBP1c and Lipogenic Gene Expressions in Hepatocytes Independently of HIF1A

**DOI:** 10.3390/cimb46030151

**Published:** 2024-03-13

**Authors:** Yong Seong Kwon, Ye Eun Cho, Yeonsoo Kim, Minseob Koh, Seonghwan Hwang

**Affiliations:** 1 College of Pharmacy and Research Institute for Drug Development, Pusan National University, Busan 46241, Republic of Korea; 2 Department of Chemistry, Pusan National University, Busan 46241, Republic of Korea

**Keywords:** dimethyloxalylglycine, prolyl hydroxylase domain, hypoxia-inducible factor-1-alpha, fatty liver, fatty acid synthesis, sterol regulatory element-binding protein-1c

## Abstract

Dimethyloxalylglycine (DMOG) is a representative inhibitor of the prolyl hydroxylase domain (PHD), which mediates the degradation of hypoxia-inducible factor-1-alpha (HIF1A). DMOG exerts its pharmacological effects via the canonical pathway that involves PHD inhibition; however, it remains unclear whether DMOG affects lipogenic gene expression in hepatocytes. We aimed to elucidate the effects of DMOG on sterol regulatory element-binding protein-1c (SREBP1c), a master regulator of fatty acid synthesis in hepatocytes. DMOG treatment inhibited SREBP1c mRNA and protein expression in HepG2 and AML12 hepatocytes and reduced the transcript levels of SREBP1c-regulated lipogenic genes. A luciferase reporter assay revealed that DMOG inhibited the transcriptional activity of SREBP1c. Moreover, DMOG suppressed SREBP1c expression in mice liver. Mechanistically, treatment with DMOG enhanced the expression of HIF1A and insulin-induced gene 2 (INSIG2), which inhibits the activation of SREBP1c. However, *HIF1A* or *INSIG2* knockdown failed to reverse the inhibitory effect of DMOG on SREBP1c expression, suggesting a redundant role of HIF1A and INSIG2 in terms of repressing SREBP1c. DMOG did not function through the canonical pathway involving inhibition of SREBP1c by PHD, highlighting the presence of non-canonical pathways that mediate its anti-lipogenic effect.

## 1. Introduction

The recent surge in obesity rates has increased the prevalence of fatty liver, which is a hepatic manifestation of metabolic syndrome characterized by the aberrant accumulation of fat in the liver [1,2,3]. Approximately 30% of the adult population is affected by fatty liver, which highlights the need to develop appropriate pharmacological interventions [4,5,6]. The hepatic fat content is regulated by a combination of four processes: de novo fatty acid synthesis, fatty acid uptake from the circulation, triglyceride secretion mediated by very-low-density lipoprotein, and fatty acid beta-oxidation [7]. Under obesogenic conditions, the balance between these four processes is tipped toward accelerated hepatic fat accumulation. Enhanced de novo fatty acid synthesis is the primary factor that causes fatty liver development in humans [8].

Sterol regulatory element-binding protein-1c (SREBP1c), a master regulator of lipogenesis, activates the transcription of various genes involved in de novo fatty acid synthesis [9]. The precursor form of SREBP1c is translated and binds to the endoplasmic reticulum (ER) membrane. SREBP1c activation requires the escort protein, SREBP cleavage activating protein (SCAP), which mediates the transport of SREBP1c from the ER to the Golgi apparatus [10]. In the Golgi apparatus, the SREBP1c precursor is subjected to proteolytic cleavage, and transcriptionally active domains migrate from the membrane to the nucleus, where SREBP1c binds to the sterol regulatory element (SRE) in the promoter and enhancer regions of its lipogenic target genes to promote their transcription [11,12]. The target genes of SREBP1c include fatty acid synthase (*FASN*), acetyl-CoA carboxylase (*ACACA*), stearoyl-CoA desaturase (*SCD*), diacylglycerol acyltransferase (*DGAT*), and glycerol-3-phosphate acyltransferase (*GPAT*), which are involved in the synthesis of fatty acid and triglyceride [13,14]. In addition to SREBP1c, other forms of SREBPs include SREBP1a and SREBP2. SREBP2 transactivates cholesterol biosynthesis genes, and SREBP1a activates the biosynthesis of both cholesterol and fatty acids, whereas SREBP1c is the major lipogenic transcription factor in the liver [11]. Insulin-induced gene-1 (INSIG1) and insulin-induced gene-2 (INSIG2) are ER membrane-anchored proteins that play important roles in regulating SREBP activity [15]. INSIG1 and INSIG2 bind to SCAP and its associated SREBPs to prevent their release from the ER membrane, thereby preventing their nuclear translocation and transactivation.

Hypoxia-inducible factor-1-alpha (HIF1A) is an oxygen-sensitive transcription factor that regulates diverse hypoxia-responsive genes, including vascular endothelial growth factor (VEGF) and glucose transporter 1. Under normal oxygen conditions, HIF1A is hydroxylated by prolyl hydroxylase domain (PHD) proteins, which promote the proteasomal degradation of HIF1A. In contrast, hypoxia inhibits PHDs, thereby stabilizing HIF1A expression. We previously reported that the promoter region of *INSIG2* includes hypoxia response elements, and *INSIG2* is a confirmed target gene of HIF1A [16]. In addition, we reported that dimethyloxalylglycine (DMOG), a PHD inhibitor, activated HIF1A and promoted INSIG2 expression in SV-589 human skin fibroblasts [16]. The upregulation of INSIG2 results in (1) the accelerated degradation of 3-hydroxy-3-methylglutaryl coenzyme A reductase (HMGCR), a rate-limiting enzyme in cholesterol biosynthesis, and (2) the inhibition of SREBP2-mediated HMGCR transcription [16]. The inhibition of HMGCR by the HIF1A-INSIG2 pathway may facilitate cellular adaptation to hypoxic conditions by suppressing cholesterol biosynthesis, a highly oxygen-consuming process that requires 11 molecules of O_2_ [17].

Considering the similarities in the regulatory mechanisms of SREBP1 and SREBP2 mediated by INSIG2, in this study, we aimed to investigate the effects of DMOG on the expression of SREBP1c and lipogenic genes in hepatocytes. Herein, we provide experimental evidence that DMOG can inhibit the processing and nuclear expression of SREBP1c, suppress SRE promoter activity, and reduce the transcription of SREBP1c target genes and SREBP1c itself. Moreover, we observed that DMOG exerted an inhibitory effect on the expression of SREBP1c in the liver of mice. Our mechanistic studies revealed that DMOG could stabilize HIF1A and upregulate INSIG2 in hepatocytes; however, this effect was not reversed in the loss-of-function studies of HIF1A or INSIG2, highlighting the redundancy of the HIF1A-INSIG2 pathway in SREBP1c repression mediated by DMOG.

## 2. Materials and Methods

### 2.1. Cell Culture

HepG2 human hepatoma cells were cultured at 37 °C under 5% CO_2_ in Dulbecco’s modified Eagle’s medium (DMEM; Hyclone, Logan, UT, USA), supplemented with 10% fetal bovine serum (FBS; Hyclone) and 1% penicillin-streptomycin (Welgene, Gyeongsan, Republic of Korea). AML12 mouse hepatocytes were cultured at 37 °C under 5% CO_2_ in DMEM/F-12, 1:1 Mixture (Welgene), supplemented with 10% FBS, 1% penicillin-streptomycin, 400 μg/mL dexamethasone (TCI, Tokyo, Japan), 7.5% sodium bicarbonate (Thermo Fisher Scientific, Waltham, MA, USA), and 1% insulin-transferrin-selenium pyruvate supplement (Welgene). T-75 flasks were used for cell propagation, and the experiments were performed in 6-, 12-, and 96-well plates. The cells were grown until 80% confluency was reached and split at least twice a week.

### 2.2. Cell Lysis, Subcellular Fractionation, and Immunoblot Analysis

To obtain whole-cell lysates, cells were lysed in RIPA buffer (Enzynomics, Seongnam, Republic of Korea) containing a cocktail of protease and phosphatase inhibitors (GenDEPOT, Baker, TX, USA) according to the manufacturer’s instructions. The subcellular fractionation of cells and mouse liver by differential centrifugation was performed, as previously described [18]. The lysate protein concentration was determined using a BCA kit (Thermo Fisher Scientific, Waltham, MA, USA). Immunoblot analyses were performed, as previously described [16,19]. Briefly, the protein extracts were loaded onto 6, 8, or 12% polyacrylamide gels and transferred to nitrocellulose membranes (Thermo Fisher Scientific). The protein bands were visualized using Pierce ECL Western Blotting Substrate (Thermo Fisher Scientific) and analyzed using a ChemiDocTM MP Imaging System (Bio-Rad, Hercules, CA, USA). Antibodies against SREBP1c and GAPDH were purchased from Santa Cruz Biotechnology (Dallas, TX, USA). The antibody against calnexin was obtained from Novus Biologicals (Littleton, CO, USA). The antibody against HIF1A was procured from Bethyl Laboratories (Montgomery, TX, USA). The antibody against lamin B1 was purchased from Abcam (Cambridge, MA, USA).

### 2.3. Total RNA Isolation and Reverse Transcription-Quantitative PCR

Total RNA was purified from the cell cultures using RiboExTM reagents (Geneall, Seoul, Republic of Korea) according to the manufacturer’s instructions. A total of 1 microgram of RNA was reverse-transcribed into cDNA using a ReverTraAce cDNA synthesis kit (Toyobo, Osaka, Japan). cDNAs were amplified using an SYBR Green Kit (Enzynomics, Daejeon, Republic of Korea) on a CFX Connect Real-Time PCR System (Bio-Rad, Hercules, CA, USA). The 2^−∆∆Ct^ method was used to calculate mRNA levels. The primer sequences used for PCR are listed in Table 1.

### 2.4. Transfection and Luciferase Assays

HepG2 cells were seeded onto 96-well plates at a density of 1.5 × 10^4^ cells/well. On the following day, the cells were transfected with an LXRα-overexpressing plasmid and firefly luciferase vector containing SREs. For the control experiments, cells were transfected with pCMV and pGL3 plasmids. The cells were transfected with the TK-Renilla luciferase vector to normalize firefly luciferase activity. The plasmids have been described previously [20] and were generously provided by Prof. Sung Hwan Ki (Chosun University, Gwangju, Republic of Korea). Transfection was performed using Lipofectamine 3000 (Thermo Fisher Scientific), according to the manufacturer’s protocol. After 24 h of transfection, the cells were further treated with T0901317 (10 μM) for 12 h following a 30 min pretreatment with DMOG (0.5 or 1 mM). T0901317 and DMOG were purchased from MedChemExpress (Monmouth Junction, NJ, USA). Firefly and Renilla luciferase activities in cell lysates were measured using the Dual-Glo Luciferase Assay System (Promega, Madison, WI, USA) according to the manufacturer’s protocol, using a microplate reader (Tecan, Mannedorf, Switzerland).

### 2.5. RNA Interference

Human *INSIG2* siRNA (ID: s226716) was purchased from Thermo Fisher Scientific. *GFP* siRNA (SP-2012) was purchased from Bioneer (Daejeon, Republic of Korea). Human *HIF1A* siRNA was designed and synthesized by Bioneer (sequence: 5′-CAGAAAUGGCCUUGUGAAAUU-3′) [16]. HepG2 cells were seeded in 6-well plates and cultured for less than 24 h until they reached 80% confluency. The cells were transfected with Lipofectamine RNAiMax (Thermo Fisher Scientific) for 24 h and treated with DMOG for the indicated time periods.

### 2.6. Animal Studies

Seven-week-old male C57BL/6J mice were obtained from Hyochang Science (Daegu, Republic of Korea). The mice were administered DMOG dissolved in saline via intraperitoneal injection at a dose of 320 mg/kg/day for 3 consecutive days. The control mice received the saline vehicle. At the end of the treatment period, the mice were sacrificed, and their livers were harvested, snap-frozen, and stored in liquid N_2_ until analysis. All mice were housed in colony cages under a 12 h light-dark cycle and fed a chow diet ad libitum. Animal experiments were performed following the National Institutes of Health Guidelines for the Care and Use of Laboratory Animals, using the protocols approved by the Pusan National University Animal Care and Use Committee (approval number: PNU-2023-0376).

### 2.7. Statistical Analysis

The data are expressed as the mean ± standard error of the mean. Data analyses were performed using GraphPad Prism software (v. 8.0a; GraphPad Software, Inc., La Jolla, CA, USA). The Student’s *t*-test was used to compare the values obtained from the two groups. A one-way analysis of variance was conducted to compare more than three groups, followed by a post-hoc Tukey test to determine specific group differences. Statistical significance was set at *p* < 0.05.

## 3. Results

### 3.1. DMOG Represses SREBP1c Expression in Hepatocytes

**Figure 1:**
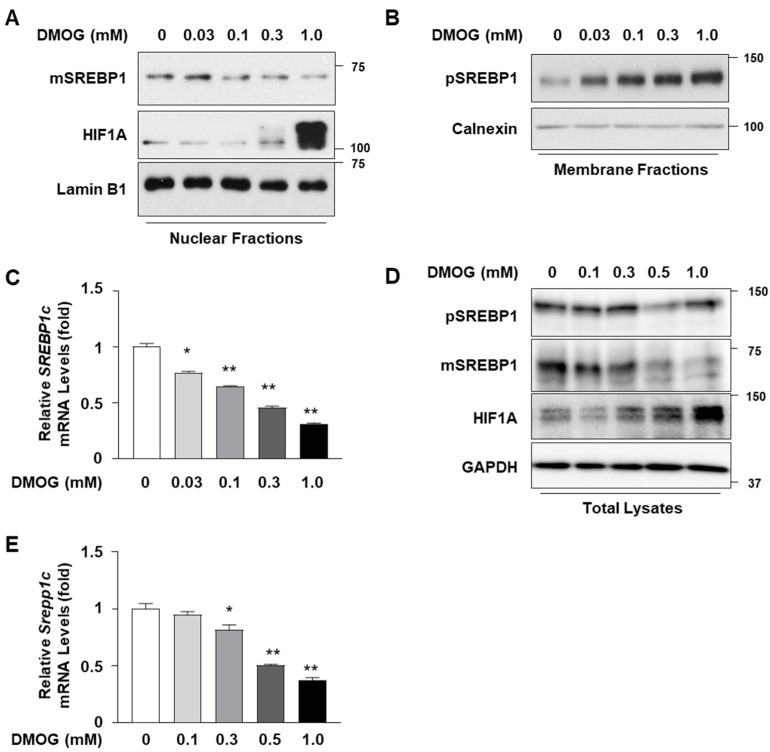
DMOG represses the expression of SREBP1c in HepG2 and AML12 cells. (**A**–**C**) HepG2 cells were treated with increasing concentrations of DMOG for 24 h. Nuclear and membrane extracts were subjected to immunoblot analyses for SREBP1, HIF1A, lamin B1, and calnexin (panels (**A**,**B**)), and RNA extracted from cells was used for RT-qPCR analyses for *SREBP1c* (panel (**C**)). *RPLP0* was used as a reference gene for RT-qPCR analyses. (**D**,**E**) AML12 cells were treated with increasing concentrations of DMOG for 24 h. Total cell lysates were subjected to immunoblot analyses of SREBP1, HIF1A, and GAPDH (panel (**D**)), and RNA extracted from cells was used for RT-qPCR analyses for *Srebp1c* (panel (**E**)). *Rplp0* was used as a reference gene for RT-qPCR analyses. The values represent the mean ± standard error of the mean. For statistical analysis, a one-way analysis of variance was conducted, followed by post-hoc Tukey tests to determine specific group differences (* *p* < 0.05; ** *p* < 0.01). DMOG: dimethyloxalylglycine; pSREBP1: precursor form of SREBP1 (125 kDa in size); mSREBP1: mature form of SREBP1 (68 kDa in size); RT-qPCR: reverse transcription-quantitative PCR.

In order to determine the effects of DMOG on the expression of SREBP1c in hepatocytes, the HepG2 cells were treated with increasing concentrations of DMOG. Treatment with DMOG reduced the expression of the mature form of SREBP1c in the nucleus in a concentration-dependent manner (Figure 1A). Although the anti-SREBP1 antibody recognized both SREBP1a and SREBP1c, the bands mostly represented SREBP1c, the major form expressed in hepatocytes [20]. The effect of DMOG was confirmed by the concentration-dependent increase in HIF1A expression. In contrast to the decrease in the mature form of SREBP1c, treatment with DMOG increased SREBP1c precursor levels in the membrane extracts (Figure 1B). This suggests the retention of the SCAP-SREBP1c complex in the membrane and the inhibition of SREBP1c nuclear translocation. In addition, we examined the effects of DMOG on the transcript levels of *SREBP1c* in HepG2 cells. Treatment with DMOG reduced *SREBP1c* mRNA levels in a concentration-dependent manner (Figure 1C). Similar experiments were conducted using AML12 mouse hepatocytes. Treatment with DMOG reduced the expression of the SREBP1c mature form and enhanced HIF1A expression in a concentration-dependent manner (Figure 1D). DMOG treatment reduced the transcript levels of *SREBP1c* in AML12 cells (Figure 1E). These results indicate that DMOG inhibits the transcription and expression of SREBP1c in hepatocytes.

### 3.2. DMOG Inhibits the Expression of SREBP1-Regulated Lipogenic Genes

**Figure 2:**
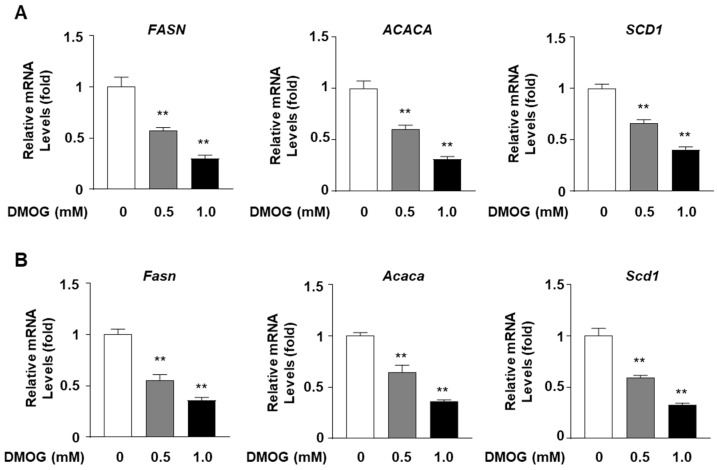
DMOG suppresses the transcription of lipogenic genes in HepG2 and AML12 cells. (**A**) HepG2 cells were treated with 0.5 and 1.0 mM DMOG for 12 h. (**B**) AML12 cells were treated with 0.5 and 1.0 mM of DMOG for 24 h. For all experiments shown in panels A and B, the RNA isolated from the cells was used for RT-qPCR for the lipogenic genes. *RPLP0* and *Rplp0* were used as the reference genes for RT-qPCR analyses. The values represent the mean ± standard error of the mean. For statistical analysis, a one-way analysis of variance was conducted, followed by post-hoc Tukey tests to determine specific group differences (** *p* < 0.01). DMOG: dimethyloxalylglycine; RT-qPCR: reverse transcription-quantitative PCR.

Given the inhibitory effect of DMOG on SREBP1c expression, we examined whether DMOG inhibits the expression of lipogenic genes, the transcription of which is governed by SREBP1c. We assessed the effect of DMOG on the transcription of three target genes of SREBP1c: *FASN*, *ACACA*, and *SCD1* [13,21,22,23]. Based on the above finding that DMOG could markedly inhibit SREBP1c expression at concentrations exceeding 0.5 mM, HepG2 cells were treated with 0.5 and 1.0 mM DMOG for 24 h. DMOG treatment reduced the mRNA levels of *FASN*, *ACACA*, and *SCD1* in a dose-dependent manner (Figure 2A). Similarly, 24 h treatment with DMOG suppressed the transcription of *Fasn*, *Acaca*, and *Scd1* in AML12 cells (Figure 2B). These findings indicate that DMOG could repress the transcription of SREBP1c-downstream lipogenic genes in hepatocytes.

### 3.3. DMOG Inhibits the Transcriptional Activity of SREBP1c

**Figure 3:**
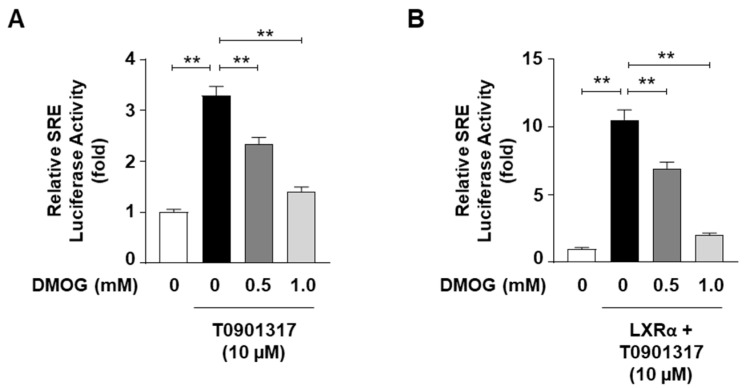
DMOG inhibits the SRE-luciferase activity. (**A**) HepG2 cells were transfected with an SRE-containing luciferase vector (50 ng) and treated with T0901317 (10 μM) following a 30 min pretreatment with DMOG (0.5 and 1.0 mM). (**B**) HepG2 cells were transfected with an SRE-containing luciferase vector (50 ng) and a vector overexpressing LXRα (50 ng). The cells were concomitantly treated with T09013137 following a 30 min pretreatment with DMOG (0.5 and 1.0 mM). After incubation for 24 h, firefly luciferase activity, which represents SRE reporter activity, was measured and normalized against Renilla luciferase activity using the Dual-Glo Luciferase Assay System. The values represent the mean ± standard error of the mean. For statistical analysis, a one-way analysis of variance was conducted, followed by post-hoc Tukey tests to determine specific group differences (** *p* < 0.01). DMOG: dimethyloxalylglycine.

Given the ability of DMOG to reduce the mRNA levels of SREBP1c target genes, we next determined whether DMOG could suppress the transcriptional activity of SREBP1c. HepG2 cells were transfected with a luciferase vector containing SREs as reporter genes to assess the transcriptional activity of SREBP1c. The treatment of HepG2 cells with T0901317, an agonist of the liver X receptor (LXR) that stimulates SREBP1c expression, enhanced SRE-containing promoter activity by approximately 3.5-fold. Pretreatment with DMOG suppressed luciferase activity in a concentration-dependent manner (Figure 3A). Transfection with the LXR-overexpressing vector, in addition to T0901317 treatment, further enhanced SRE-luciferase activity by approximately 10-fold, which was significantly reduced by DMOG (Figure 3B). These results indicate that DMOG could repress the transcriptional activity of SREBP1c, which may explain the mechanism underlying the inhibition of SREBP1c target gene transcription mediated by DMOG.

### 3.4. Inhibitory Effect of DMOG on SREBP1c Expression Is Independent of HIF1A and INSIG2

We have previously reported that HIF1A transactivates INSIG2 and that DMOG increases INSIG2 expression through HIF1A activation in SV-589 human skin fibroblasts [16]. As INSIG2 binds to the SCAP-SREBP1c complex and hinders the proteolytic processing of SREBP1c, we sought to determine whether the DMOG-induced inhibition of SREBP1c is mediated via HIF1A and INSIG2.

**Figure 4:**
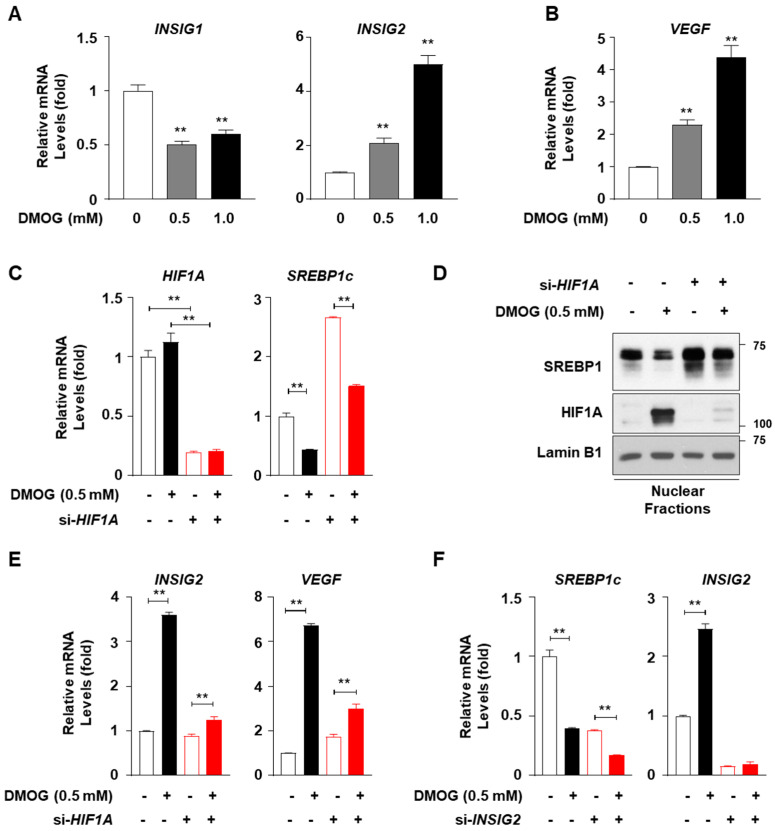
Knockdown of *HIF1A* or *INSIG2* does not abolish the DMOG-mediated inhibition of SREBP1c in HepG2 cells. (**A**,**B**) HepG2 cells were treated with DMOG (0.5 and 1.0 mM) for 12 h. RNA isolated from the cells was subjected to RT-qPCR analyses for *INSIG1*, *INSIG2*, and *VEGF*. (**C**–**E**) HepG2 cells were transfected with siRNA (30 pmol) against *GFP* or *HIF1A* for 24 h and treated with DMOG (0.5 mM) for an additional 24 h. RNA isolated from the cells was used for RT-qPCR analyses for *HIF1A*, *SREBP1c*, *INSIG2*, and *VEGF* (panels (**C**,**E**)). The nuclear extracts of the cells were subjected to immunoblot analyses for SREBP1, HIF1A, and lamin B1 (panel (**D**)). (**F**) HepG2 cells were transfected with siRNA (10 pmol) against *GFP* or *INSIG2* for 24 h and treated with DMOG (0.5 mM) for an additional 24 h. The RNA extracted from cells was used for RT-qPCR analyses for *SREBP1c* and *INSIG2*. *RPLP0* was used as the reference gene for RT-qPCR analyses. The values represent the mean ± standard error of the mean. For statistical analysis, a one-way analysis of variance was conducted, followed by post-hoc Tukey tests to determine specific group differences (** *p* < 0.01). DMOG: dimethyloxalylglycine; RT-qPCR: reverse transcription-quantitative PCR.

Treatment with DMOG increased *INSIG2* mRNA levels in HepG2 cells, similar to our previous observations in SV-589 cells [16], whereas DMOG reduced the mRNA levels of *INSIG1*, another inhibitor of SREBP1c nuclear translocation (Figure 4A). The DMOG-dependent activation of HIF1A was confirmed by the enhanced mRNA levels of *VEGF*, a representative target gene of HIF1A (Figure 4B). In order to verify whether DMOG inhibited SREBP1c through HIF1A activation, HepG2 cells were subjected to a loss-of-function study for HIF1A using siRNAs against *HIF1A* (si-*HIF1A*). Transfection with si-*HIF1A* reduced the mRNA levels of *HIF1A* by approximately 80% (Figure 4C). DMOG reduced the *SREBP1c* mRNA levels in HepG2 cells transfected with a control siRNA against *GFP* (si-*GFP*). *HIF1A* knockdown increased *SREBP1c* mRNA levels by approximately 2.5-fold. The ability of DMOG to lower *SREBP1c* mRNA levels remained intact even in si-*HIF1A*-transfected cells (Figure 4C). Likewise, at the protein level, *HIF1A* knockdown did not interfere with the ability of DMOG to repress SREBP1 expression in the nuclei (Figure 4D). In order to verify the functional integrity of si-*HIF1A* transfection, we further examined the transcription of HIF1A target genes, such as *INSIG2* and *VEGF*. The DMOG-dependent induction of *INSIG2* and *VEGF* was reversed by transfection with si-*HIF1A* (Figure 4E), suggesting the effective knockdown of *HIF1A* and the diminished upregulation of HIF1A target genes. Therefore, these results indicate that although DMOG stabilizes HIF1A, its ability to repress SREBP1c is independent of HIF1A.

In order to investigate the involvement of INSIG2 in the DMOG-mediated inhibition of SREBP1c, we performed loss-of-function studies on INSIG2 in HepG2 cells using siRNA-mediated *INSIG2* knockdown. Transfection with siRNA against *INSIG2* (si-*INSIG2*) reduced *INSIG2* mRNA levels by approximately 90% (Figure 4F). Transfection with si-*INSIG2* did not suppress the ability of DMOG to inhibit *SREBP1c* transcription. These results suggest that DMOG could inhibit SREBP1c through a mechanism independent of HIF1α-mediated INSIG2 upregulation.

### 3.5. DMOG Reduces SREBP1c Expression in Mice Liver

**Figure 5:**
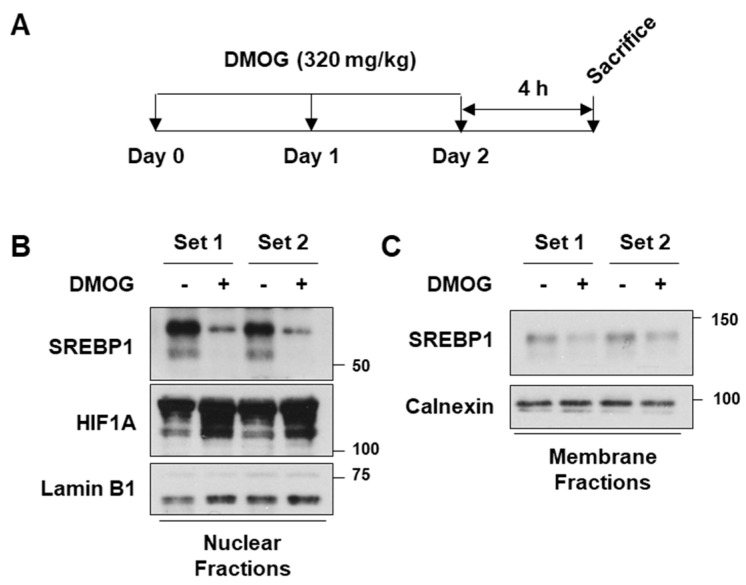
DMOG inhibits SREBP1c expression in the liver of mice. (**A**) Male C57BL/6J mice (*n* = 4 and 7 weeks of age) were intraperitoneally injected with DMOG (320 mg/kg) or saline once daily for 3 days. Four hours after the final injection, the mice were sacrificed. (**B**,**C**) The livers were homogenized, and the homogenates were centrifuged to obtain the nuclear and membrane fractions. The nuclear extracts were subjected to immunoblot analyses for SREBP1c, HIF1A, and lamin B1, while the membrane extracts were blotted for SREBP1c and calnexin. The animal experiments were performed twice, each of which was labeled as Set 1 and Set 2, respectively. In each experiment, the protein extracts from four individual samples were pooled for immunoblot analyses. DMOG: dimethyloxalylglycine; RT-qPCR: reverse transcription-quantitative PCR.

Based on the observation that DMOG repressed SREBP1c and its downstream lipogenic genes, we next determined the in vivo efficacy of DMOG. Male C57BL/6J mice were administered DMOG (320 mg/kg) once daily for 3 days (Figure 5A). Treatment with DMOG enhanced HIF1A expression and reduced the expression of the mature form of SREBP1c in the nuclear extracts of liver tissues (Figure 5B). Similarly, treatment with DMOG reduced the precursor form of SREBP1c in the membrane fractions of liver tissues (Figure 5C). These findings emphasize the effectiveness of DMOG in suppressing SREBP1c expression in mice liver.

## 4. Discussion

DMOG has been shown to reduce SREBP1c expression in SV-589 skin fibroblasts [16]. However, the effects of DMOG and its underlying mechanisms remain poorly characterized in hepatocytes, where SREBP1c functions as a master regulator of lipogenesis. This study expands on previous findings by demonstrating that DMOG inhibits SREBP1c and lipogenic genes in hepatocytes and mice liver. This notion is supported by several lines of evidence showing that DMOG administration inhibits SREBP1c expression at the transcript and protein levels as well as its transcriptional activity, resulting in the downregulation of its lipogenic target genes such as *FASN*, *ACACA*, and *SCD1*. Moreover, we revealed that DMOG could increase the expression of HIF1A and INSIG2 in hepatocytes. However, the knockdown of either *HIF1A* or *INSIG2* could not reverse the inhibitory effect of DMOG on SREBP1c expression.

The failure of *HIF1A* or *INSIG2* knockdown to reverse the effect of DMOG suggests several possible mechanisms through which DMOG represses SREBP1c and lipogenic genes. First, DMOG probably does not function through HIF1A activation to inhibit SREBP1c processing or lipogenic gene induction. DMOG is an analog of 2-oxoglutarate (2-OG); thus, it competes with 2-OG binding to PHD [24,25]. DMOG activates HIF1A by inhibiting PHD, which belongs to the 2-OG-dependent dioxygenase (2-OGDD) superfamily [26]. The enzymatic function of each 2-OGDD, including the PHDs, requires 2-OG, molecular oxygen, and Fe^2+^ [27,28]. Therefore, DMOG may interrupt the function of PHDs and other enzymes in the 2-OGDD superfamily. 2-OGDDs participate in various biological processes, including the epigenetic regulation of gene transcription, the metabolic reprogramming of cells, and extracellular matrix formation [29], all of which are potentially affected by the administration of DMOG. Therefore, DMOG-dependent changes in SREBP1c activity are likely mediated by a non-PHD member of the 2-OGDD superfamily or an unexplored target of DMOG outside the 2-OGDD superfamily. DMOG administration (0.1 mM) reduced the mRNA and protein levels of SREBP1c in HepG2 cells, although expression of HIF1A was not induced at this concentration (Figure 1A,C), suggesting the presence of an unidentified mediator that responds to DMOG even at low concentrations, without HIF1A activation. Among the numerous potential regulators of SREBP1c, AMPK has drawn our attention because it activates AMP-activated protein kinase (AMPK) signaling in cardiomyocytes [30] and inhibits SREBP1c expression through Ser372 phosphorylation [31]. Future investigations into the involvement of AMPK in SREBP1c phosphorylation will provide a better understanding of the mechanisms underlying the effects of DMOG on SREBP1c.

In addition, it is conceivable that the HIF1A-INSIG2 pathway partially contributes to the effect of DMOG on SREBP1c, albeit with redundant capacity. INSIG1 and INSIG2 share approximately 60% of amino acid sequence identity and have been reported to be functionally redundant [32,33,34]. Considering the regulation of SREBP1c by INSIGs in the liver, Engelking et al. reported that the deletion of either *INSIG1* or *INSIG2* failed to increase the expression of SREBP1 in mice liver, whereas the concomitant deletion of the two genes markedly enhanced the hepatic expression of SREBP1 [35]. Therefore, the remaining INSIG1 may contribute to the ability of DMOG to suppress SREBP1c expression in hepatocytes knocked down with si-*INSIG2*. Moreover, one limitation of this study is the potential incompleteness of the loss-of-function achieved by si-*HIF1A* and si-*INSIG2*. A more definitive assessment of whether the impact of DMOG on SREBP1c operates independently of the HIF1A-INSIG2 pathway could be established by employing stable cell lines deficient in *HIF1A* or *INSIG2*.

Elevated levels of INSIG2 may not fully inhibit SREBP2 owing to the tight feedback regulation of cholesterol homeostasis. The DMOG-induced activation of the HIF1A-INSIG2 pathway inhibits SREBP2 and HMGCR, which may consequently suppress cholesterol synthesis in cells. A decrease in sterol levels could result in the compensatory disinhibition of SREBPs by preventing the interaction between INSIG2 and the SCAP-SREBP complex, which could override the ability of DMOG to inhibit SREBP1c via HIF1A-mediated INSIG2 upregulation.

Despite the logical assumption that hypoxia and HIF1A activation downregulate SREBP2 to mitigate oxygen-demanding cholesterol biosynthesis and conserve oxygen for essential cellular functions, the role of HIF1A in the regulation of fatty acid synthesis remains debatable. Nishiyama et al. showed that the hepatocyte-specific deletion of *Hif1a* could aggravate alcohol-induced fatty liver in mice, thereby suggesting that HIF1A exerts a protective function against fat deposition [36]. Conversely, Nath et al. reported conflicting observations: while the hepatocyte-specific expression of the constitutively active form of *Hif1a* increased fat accumulation in mice, hepatocyte-specific *Hif1a* deletion attenuated fatty liver [37]. Given the discordant findings regarding the involvement of HIF1A in SREBP1c regulation, it is conceivable that DMOG may inhibit SREBP1c irrespective of HIF1A.

In order to elucidate the pharmacological effect of DMOG on hepatocytes, this study delineated its inhibitory effects on SREBP1c and lipogenic genes while acknowledging the intricate mechanisms underlying its action. The growing prevalence of metabolic syndrome and fatty liver disease provides a compelling rationale for future research endeavors to scrutinize the molecular components involved in the anti-lipogenic properties of DMOG. These findings may lead to the discovery of molecular targets amenable to therapeutic interventions for fatty liver disease.

## Figures and Tables

**Table 1:** Primer sequences for RT-qPCR.

Species	Gene	Forward (5′-3′)	Reverse (5′-3′)
Human	*RPLP0(36B4)*	CGACCTGGAAGTCCAACTAC	ATCTGCTGCATCTGCTTG
Human	*SREBP1c*	TCGCGGAGCCATGGATT	GGAAGTCACTGTCTTGGTTGTTGA
Human	*FASN*	TCGTGGGCTACAGCATGGT	GCCCTCTGAAGTCGAAGAAGAA
Human	*ACACA*	TTCAGAGGCAGGGTGGGTTA	ACATACTCGTTTGTGTCATAATTTGGT
Human	*SCD1*	TCACCACATTCTTCATTGATTGC	TTGGAGACTTTCTTCCGGTCAT
Human	*INSIG1*	CCCAGATTTCCTCTATATTCGTTCTT	CACCCATAGCTAACTGTCGTCCTA
Human	*INSIG2*	TGTCTCTCACACTGGCTGCACTA	CTCCAAGGCCAAAACCACTTC
Human	*VEGF*	CGCAGCTACTGCCATCCAAT	TGGCTTGAAGATGTACTCGATCTC
Human	*HIF1A*	ATCCATGTGACCATGAGGAAATG	TCGGCTAGTTAGGGTACACTTC
Mouse	*Rplp0(36B4)*	AGATTCGGGATATGCTGTTGGC	TCGGGTCCTAGACCAGTGTTC
Mouse	*Srebp1c*	GGAGCCATGGATTGCACATT	GGCCCGGGAAGTCACTGT
Mouse	*Fasn*	GCTGCGGAAACTTCAGGAAAT	AGAGACGTGTCACTCCTGGACTT
Mouse	*Acaca*	TGGACAGACTGATCGCAGAGAAAG	TGGAGAGCCCCACACACA
Mouse	*Scd1*	CCGGAGACCCCTTAGATCGA	TAGCCTGTAAAAGATTTCTGCAAACC

## Data Availability

The data presented in this study are available upon request from the corresponding author.
